# Mediating effects of positive self-beliefs, social emotions, and worry on childhood socioeconomic status and prosocial and antisocial rule-breaking

**DOI:** 10.1038/s41598-025-20845-6

**Published:** 2025-10-23

**Authors:** Xiaoning Zhang, Shiqi Yu, Xuejiao Zhu, Xinru Huang, Youhua Chen

**Affiliations:** 1 School of Public Health and Nursing, Hangzhou Normal University, Yuhangtang Road, Yuhang District, Hangzhou, 2318, 311121 Zhejiang China; 2 Zhejiang Philosophy and Social Science Laboratory for Research in Early Development and Childcare, Hangzhou Normal University, Hangzhou, 311121 Zhejiang China; 3 School of Management, Xuzhou Medical University, 209 Tongshan Road, Xuzhou, 221004 China; 4 Humanities School, Southeast University, 2, Southeast University Road, Nanjing, 211102 China

**Keywords:** Positive self-beliefs, Social emotions, Worry, Childhood socioeconomic status, Prosocial behaviors, Psychology, Human behaviour

## Abstract

Examining the impact of childhood socioeconomic status (SES) on prosocial and antisocial behaviors through positive self-beliefs, social emotions, and worry could be critical for intervention strategies. This study collected data, including sociodemographic characteristics, childhood socioeconomic status, the Oxford positive self, the Dunn Worry Questionnaire, social anxiety scale for social media users, prosocial and antisocial rule-breaking, and social–emotional expertise in eastern China. Structural equation modeling (SEM) was employed to scrutinize pathways from childhood SES to prosocial and antisocial behaviors through positive self-beliefs, social emotions, and worry. A total of 482 adolescents, mean age was 18.58 months (SD = 1.11). Childhood SES significantly influenced prosocial and antisocial behaviors through positive self-beliefs and worry. Childhood SES significantly influenced prosocial and antisocial behaviors through social emotions, positive self-beliefs and worry. Childhood SES significantly influenced prosocial and antisocial behaviors through social emotions and worry. Childhood SES significantly influenced prosocial and antisocial behaviors through worry. The findings highlight the need for intervention programs in upper- and middle-income countries (UMICs) that aim to improve prosocial behaviors by fostering childhood SES through positive self-beliefs, social emotions, and worry.

## Introduction

Prosocial behavior refers to actions intended to benefit others, while antisocial behavior involves harmful, hostile actions; individuals can exhibit both at varying levels, indicating prosocial and antisocial behaviors on separate dimensions^1^. Rule-breaking is typically viewed as a subset of antisocial behavior, a distinct category of rule-breaking that aims to benefit others, which has not been given adequate research attention despite its significant historical and societal implications^2^. Prosocial rule-breaking aims to help others without regard for oneself, while antisocial rule-breaking aims to benefit oneself without regard for others, though both involve violating institutional rules^1^. Prosocial behavior is linked to moral identity^3^, empathy, and the tendency to experience moral emotions^1^. A strong moral identity integrates internalized moral values into individual’ self-concept guiding behaviors^4^.

Mental health conditions emerge from the interplay of multiple factors that extend beyond diagnostic boundaries, with worry identified as a plausible contributory factor as evidenced by longitudinal data from a British National Survey^5^. Excessive worry is identified linked to the development of mental health disorders, including anxiety^6^, depression^7^ and eating disorders^8^. Worry introduces and maintains fearful thoughts, elaborates on their content, and escalates distress by increasing the perceived likelihood of feared outcomes^9^. Problematic worry consists of persistent contemplation of issues that induce anxiety about the future, an emphasis on potential negative outcomes, catastrophizing problems, a belief in the lack of control over the thought process, and interference with daily activities and well-being^9^.

According to self-determination theory (SDT), children are more likely to sustain prosocial behaviors when their basic needs are met, and they perceive their actions and goals as self-determined and aligned with their intrinsic values^10^. Beliefs about achieving goals, performing tasks effectively, and succeeding are associated with behavioral activation, and mastery control techniques^11^. Beliefs concerning coping, perseverance, and resilience are linked to behavioral experiments in challenging situations^12^.The dual continua model hypothesis posits that psychological well-being and mental disorders are distinct but correlated dimensions, negative self-beliefs are likely associated with fewer positive self-beliefs^13^. Positive self-beliefs, although less frequently studied, are primarily explored within the field of positive psychology as contributors to psychological well-being^14^. Positive self-beliefs highlights potentially modifiable cognitions is crucial for enhancing psychological well-being^13^. The enjoyment of activities and the ability to relax correspond to savoring and relaxation techniques, while positive self-beliefs align with identifying strengths and values^15^.

The biological embodiment of infants not only links to the social environment but also generates the social-emotional context necessary for their development, as their need for care fosters these interactions^16^. Prosocial and antisocial behaviors are significantly influenced by social emotions, with emotional rejection amplifying aggressive tendencies while diminishing prosocial behaviors^17^. Engaging in prosocial behaviors necessitate socio-cognitive skills, including the capacity for effective emotion regulation^18^. Social–Emotional Expertise (SEE) refers to the ability to effectively coordinate affective gestures and vocalizations while understanding the connection between prosocial cognition and behavior^19^. SEE is characterized by an automatic response to social cues, allowing individuals to navigate social emotions, such as empathy and moral emotions (e.g., shame, guilt), with minimal conscious effort^20^.

**Fig. 1:**
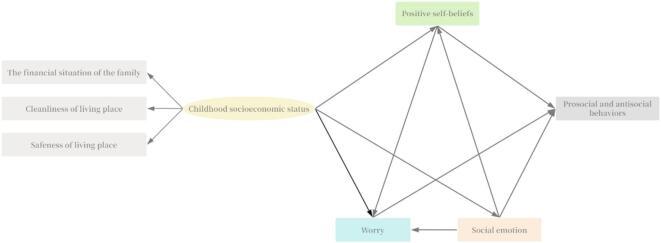
Conceptual framework and hypotheses.

Moffitt’s theoretical model proposes two distinct developmental pathways for antisocial behaviors, life-course-persistent, characterized by early-onset aggression continuing into midlife^21^. The adolescence-limited pathway, where temporary antisocial behaviors may emerge during adolescence and are more typical than pathological^22^. Life course epidemiology theory suggests that social and economic exposures during childhood^23^, can have long-lasting effects on health outcomes in later life^24^. Childhood socioeconomic status (SES) disparities significantly contribute to inequalities in childhood health^25^, mediating models propose that childhood SES can affect adult health^26^. Social Information Processing (SIP) theory is a key socio-cognitive model that has been applied to predict the likelihood of aggressive behaviors in parents, across different stages of child development^27^. Therefore, examining predictors of prosocial and antisocial behaviors necessitates considering the influence of childhood SES. While limited studies have examined the roles of positive self-beliefs and worry in mediating the relationship between childhood SES and prosocial and antisocial behaviors. Drawing from the World Health Organization’s 2010 report “A Conceptual Framework for Action on the Social Determinants of Health”^28^, this study outlines the key elements of the conceptual framework in the hypothesized model illustrated in Fig. 1: (1) a direct effect of disadvantaged socioeconomic status in childhood on prosocial and antisocial behaviors; and (2) the influence of disadvantaged socioeconomic status in childhood on prosocial and antisocial behaviors through the mediating factors of positive self-beliefs, social emotions, and worry.

## Methods

### Participants and procedure

The sample consisted of 482 respondents aged 18 and 20 years (mean age = 18.58 years, SD = 1.11), with 77.18% being female. Respondents were recruited from a university in eastern China between October 1, 2023, and March 1, 2024. Individuals with a history of mental illness were excluded. The sample size exceeded the requirement for mediation models, achieving a power of 0.80 for detecting small-to medium effect sizes^29^.

### Measures

#### Sociodemographic characteristics

Data collected included age, sex, residence area (urban, rural, and urban-rural fringe), and annual household income (<100,000, 100,000−150,000, >150,000 RMB).

### Prosocial and antisocial rule-breaking

The Prosocial and Antisocial Rule-Breaking (PARB) scale was designed to measure the likelihood of self-reported prosocial rule-breaking (20 items) and antisocial rule-breaking (18 items). Respondents rated the likelihood of breaking each rule on a scale from 1 (very unlikely) to 7 (very likely). The instrument demonstrated high reliability and validity, with Cronbach’s alpha of 0.81 for prosocial and 0.93 for antisocial^1^.

### Childhood socioeconomic status (SES)

Childhood SES is typically assessed using the financial situation of the family^30^, primary residence^31^, safety of the living place^32^, annual household income^33^, and cleanliness of living place^34^.

Primary residence was determined based on the question: “Where did you mainly live before the age of 16? Was it in a village, city/town, urban-rural fringe, or another type of area?” Responses were categorized into “urban”, “rural”, or “urban-rural fringe”.

The financial situation of the family was assessed by asking: “Before the age of 17, how did your family’s financial situation compare to the average family in the same community or village at that time?” Responses were rated as “a lot better off”, “somewhat better off”, “about the same”, “somewhat worse off” or “a lot worse off”.

Cleanliness of living place was evaluated with the question: “When you were a child, was the neighborhood where you lived very clean and attractive?” The responses were categorized as “very clean and attractive”, “somewhat clean and attractive”, “not very clean and attractive”, or “not clean and attractive at all”.

Safety of living place was assessed with the question: “When you were a child, how safe was it to be out alone at night in your neighborhood?” Responses were rated as “a lot better off than them”, “somewhat better off than them”, “about the same as them”, “somewhat worse off than them” or “a lot worse off than them”.

### The positive self-beliefs

The Oxford Positive Self Scale was developed to evaluate the lived experience of mental health issues by focusing on cognitions strongly linked to psychological well-being^13^. The scale is designed to identify positive self-cognitions that can be targeted through psychological interventions. Respondents were asked to evaluate their self-beliefs over the past week. Each item was rated on a scale from 0 to 4: do not believe it, believe it slightly, believe it moderately, believe it very much, and believe it totally. Higher scores indicate stronger endorsement of the items, with Cronbach’s alpha values above 0.81.

### The Dunn worry questionnaire

The Dunn Worry Questionnaire was designed to evaluate worry the frequency of worry over a one-month. Comprising 10 items, respondents rate each item on a 0–4 Likert scale (0 = None of the time, 1 = Rarely, 2 = Some of the time, 3 = Often, 4 = All of the time). This instrument demonstrated high reliability and validity, with Cronbach’s alpha exceeding 0.85^9^.

### Social–emotional expertise

Social–emotional expertise (SEE) assesses various cognitive abilities essential for social interactions, focusing on the timing and synchronization of behaviors that enhance overall social–emotional functioning. The scale consists of 26 items, each rated on a 7-point Likert scale ranging from never, neutral to always. The instrument demonstrated acceptable validity and reliability, with Cronbach’s alpha of 0.90^19^. In this study, the instrument demonstrated acceptable validity and reliability, with Cronbach’s alpha exceeding 0.7.

### Data analyses

Data analyses were conducted using SPSS 26.0 for basic descriptive statistics and preliminary analyses (IBM Corp., Armonk, NY, USA), AMOS 28.0 (IBM Corporation, Armonk, NY, USA), and R 4.1.0 (R Foundation for Statistical Computing, Vienna, Austria) with the lavaan package for Structural Equation Modeling (SEM). The datasets were meticulously double-checked for errors, ensuring completeness and accuracy with no missing values. Pearson’s correlation analysis was employed to investigate the relationships among sociodemographic characteristics, childhood SES, social emotions, positive self-beliefs, worry, and prosocial and antisocial behaviors. The correlation coefficients were interpreted as small (*r* ≥ .10), moderate (*r* ≥ .30), and strong (*r* ≥ .50).

Based on the conceptual framework, this study employed SEM to analyze the direct and indirect effects of childhood SES on prosocial and antisocial behaviors.

SEM was applied to determine the direct and indirect effects between outcome, observed variables, and latent variables. The total effect, which is the sum of the direct and indirect effects, can be mathematically expressed as^35^: c = c′ + ab, where c represents the total effect, c′ = direct effect, and ab = indirect effect. To estimate the statistical significance of the direct and indirect effects within each pathway, bias-corrected bootstrapping with 2000 bootstrap samples was employed.

SEM with maximum likelihood estimation was utilized to assess both direct and indirect pathways linking childhood SES to prosocial and antisocial behaviors, mediated by positive self-beliefs and worry. Sociodemographic variables that demonstrated significant associations with childhood SES, prosocial behaviors, positive self-beliefs, social emotions, and worry were incorporated as covariates in the SEM. Model fit was evaluated using multiple goodness-of-fit indices: the Comparative Fit Index (CFI) > 0.90, Incremental Fit Index (IFI) > 0.90, Goodness-of-fit Index (GFI) > 0.90, Root Mean Square Error of Approximation (RMSEA) < 0.08, and Standardized Root Mean Square Residual (SRMR) < 0.08^36,37^. Bootstrap resampling procedures (*n* = 2000) with bias-corrected 95% confidence interval (BC 95% CI) assessed the significance of effects^38,39^. Statistical significance was set at *p* < .05.

## Results

### Sociodemographic characteristics

Sociodemographic differences, childhood SES, positive self-beliefs, social emotions, and worry between prosocial and antisocial behaviors are presented in Table 1. Among the 482 participants, the mean age was 18.58 (SD = 1.11) years, with 110 (22.82%) being male, 207 (42.95%) residing in urban areas, and 241 (50.00%) reporting annual household income of less than 100,000 RMB. After implementing the Bonferroni correction to account for multiple comparisons, the findings remained statistically significant.

**Table 1:** Sociodemographic characteristics by prosocial and antisocial behaviors (*N* = 482).

Variables	*n* (%)	Prosocial behaviors	t/F	*p*	Antisocial behaviors	t/F	*p*
Sex *n* (%)							
Male	110 (22.82)	57.72 (22.01)	1.140	0.210	29.51 (18.59)	1.121	0.268
Female	372 (77.18)	49.59 (17.71)			25.49 (12.49)		
Residence							
Rural	195 (40.46)	49.64 (17.56)	1.334	0.039	26.39 (13.78)	0.836	0.788
Urban-rural fringe	80 (16.59)	50.33 (18.24)			25.66 (12.18)		
Urban	207 (42.95)	50.92 (20.16)			26.71 (15.32)		
Annual household income (RMB) *n* (%)							
< 100,000	241 (50.00)	50.76 (18.75)	0.716	0.966	26.28 (13.45)	1.045	0.394
100,000 − 300,000	197 (40.87)	48.92 (18.63)			26.44 (14.96)		
> 300,000	44 (9.13)	54.00 (19.69)			26.95 (15.00)		
Financial situation of the family							
A lot better off	35 (7.26)	54.91 (24.82)	1.249	0.088	30.60 (22.85)	1.496	0.017
Somewhat better off	129 (26.76)	50.42 (18.49)			26.09 (12.81)		
About the same	242 (50.21)	48.94 (18.16)			25.66 (13.29)		
Somewhat worse off	65 (13.49)	52.29 (17.41)			26.48 (12.99)		
A lot worse off	11 (2.28)	52.55 (22.16)			32.01 (18.89)		
Safety of living place							
Very safe	237 (49.17)	50.62 (19.47)	1.263	0.077	26.14 (14.44)	1.825	0.001
somewhat safe	226 (46.89)	49.32 (17.65)			25.91 (12.42)		
Not very safe	16 (3.32)	52.50 (19.52)			52.50 (19.52)		
Not safe at all	3 (0.62)	87.00 (14.18)			25.66 (12.18)		
Cleanliness of living place							
Very clean and attractive	162 (33.61)	50.73 (20.63)	0.972	0.549	26.45 (16.17)	0.866	0.739
Somewhat clean and attractive	292 (60.68)	50.29 (17.85)			26.36 (13.21)		
not very clean and attractive	26 (5.39)	48.12 (16.90)			25.88 (11.15)		
not clean and attractive at all	2 (0.42)	46.50 (37.48)			37.00 (26.87)		
Positive self-beliefs *M (SD)*	85.74 (20.53)		1.092	0.290		1.325	0.070
Worry *M (SD)*	34.88 (13.09)		1.340	0.037		1.248	0.121
Social emotion *M (SD)*	70.32 (21.95)		1.282	0.065		0.940	0.598

*M* mean, *SD* standard deviation.

### Pearson’s correlation analyses

The results of the correlation analysis between sociodemographic characteristics, childhood SES, prosocial and antisocial behaviors, social emotions, positive self-beliefs and worry are presented in Table 2.

**Table 2:** Descriptive statistics and correlations of sociodemographic characteristics, childhood SES, positive self-beliefs, worry, social emotion, prosocial and antisocial behaviors. Note. SD, standard deviation. *p < .05, **p < .01, ***p < .001.

Variables		Mean	SD	1		2		3		4		5		6		7		8		9		10		11	
1	Sex			1.00																					
2	Residence			0.06	1.00																				
3	Annual household income			0.04*	0.31***		1.00																		
4	The financial situation of the family			-0.04	-0.15***		-0.36***		1.00																
5	Safety of living place			0.10**	-0.16***		-0.18***		0.19***		1.00														
6	Cleanliness of living place			0.03	-0.17***		-0.15***		0.22***		0.433***		1.00												
7	Positive self-beliefs	85.74	20.53	-0.04	0.11**		0.01		-0.14***		-0.18***		-0.18***		1.00										
8	Worry	34.88	13.09	0.05	-0.12***		-0.07		0.02		0.15***		0.14***		-0.18***		1.00								
9	Social emotion	70.32	21.95	-0.03	-0.11**		-0.06		0.12***		0.11**		0.01		-0.25***		0.22***		1.00						
10	Prosocial behaviors	50.30	18.80	-0.07	0.03		0.02		-0.02		0.03		-0.03		0.03		0.10**		0.08*		1.00				
11	Antisocial behaviors	26.41	14.20	-0.12***	0.01		-0.01		-0.02		0.10**		0.01		-0.06		0.17***		0.07		0.53***		1.00		

### SEM analyses

**Fig. 2:**
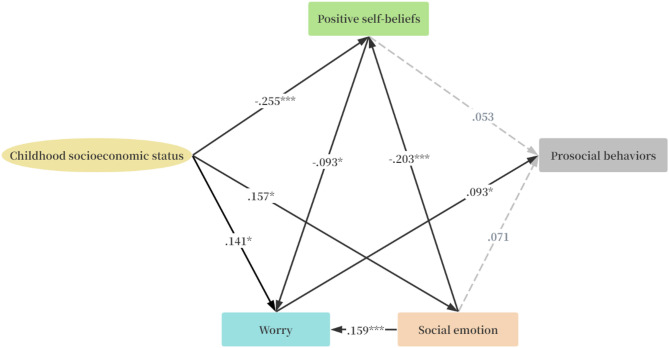
The standardized direct effects of childhood socioeconomic status on prosocial and antisocial through positive self-beliefs, worry and social emotion. (Model fit: CFI = 0.994, IFI = 0.990, GFI = 0.989, RMSEA = 0.027 and SRMR = 0.021. *p < .05, ** *p* < .01, *** *p* < .001).

**Fig. 3:**
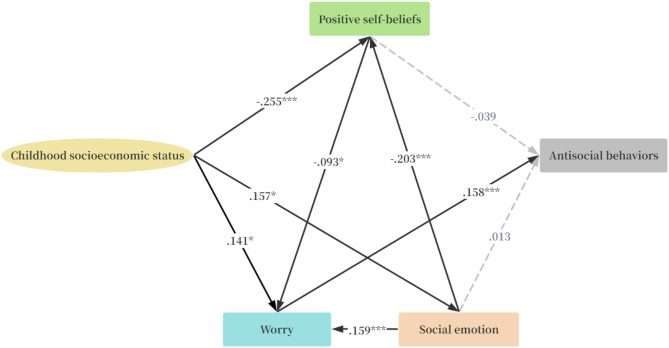
The standardized direct effects of childhood socioeconomic status on prosocial and antisocial through positive self-beliefs, worry and social emotion. (Model fit: CFI = 0.993, IFI = 0.987, GFI = 0.993, RMSEA = 0.032 and SRMR = 0.021. *p < .05, ** *p* < .01, *** *p* < .001).

Residence area of respondents was included as covariate in the SEM. The standardized path estimates illustrating the pathways from childhood SES to prosocial behaviors are shown in Fig. 2; Tables 3 and 4, SEM indicated an acceptable model fit, with CFI = 0.994, IFI = 0.990, GFI = 0.989, RMSEA = 0.035, and SRMR = 0.021. The standardized path estimates illustrating the pathways from childhood SES to antisocial behaviors are shown in Fig. 3; Tables 3 and 5, SEM indicated an acceptable model fit, with CFI = 0.993, IFI = 0.987, GFI = 0.993, RMSEA = 0.032, and SRMR = 0.021.

**Table 3:** Standardized regression weight of variables in SEM (*N* = 482).

Paths	B	β	SE	CR	*p*
**Childhood SES → Social emotion**	12.701	0.157	6.130	2.072	0.038
**Social emotion → Positive self-beliefs**	-0.189	-0.203	0.042	-4.490	< 0.001
**Childhood SES → Positive self-beliefs**	**-19.247**	**-0.255**	**5.742**	**-3.352**	**< 0.001**
**Social emotion → Excessive worry**	**0.095**	**0.159**	**0.027**	**3.475**	**< 0.001**
Positive self-beliefs → Worry	-0.059	-0.093	0.030	-1.959	0.050
**Childhood SES Worry→ Worry**	**6.803**	**0.141**	**3.388**	**2.008**	**0.045**
Positive self-beliefs → Prosocial behaviors	0.049	0.053	0.043	1.143	0.253
**Worry → Prosocial behaviors Worry**	**0.134**	**0.093**	**0.067**	**2.002**	**0.045**
Social emotion → Prosocial behaviors	0.061	0.071	0.040	1.516	0.130
**Childhood SES → Social emotion**	**0.157**	**12.701**	**0.082**	**2.072**	**0.038**
**Social emotion → Positive self-beliefs**	**-0.203**	**-0.189**	**0.061**	**-4.490**	**< 0.001**
**Childhood SES → Positive self-beliefs**	**-0.255**	**-19.247**	**0.067**	**-3.352**	**< 0.001**
**Social emotion → Worry**	**0.159**	**0.095**	**0.065**	**3.475**	**< 0.001**
Positive self-beliefs → Worry	-0.093	-0.059	0.058	-1.959	0.050
**Childhood SES→ Worry**	**0.141**	**6.803**	**0.074**	**2.008**	**0.045**
Positive self-beliefs → Antisocial behaviors	-0.039	-0.027	0.045	-0.863	0.388
**Worry → Antisocial behaviors**	**0.158**	**0.171**	**0.047**	**3.475**	**< 0.001**
Social emotion → Antisocial behaviors	0.013	0.009	0.047	0.291	0.771

Note. SEM, structural equation modeling; SES, socioeconomic status; *CR*, *critical ratio*; *SE*, standard error.

**Table 4:** Standardized direct and indirect effects from childhood SES to prosocial behaviors.

Paths	β	SE	BC 95% CI	*p*
Childhood SES → Positive self-beliefs → Worry → Prosocial behaviors	0.153	0.135	0.005 to 0.559	0.041
Childhood SES → Social emotion → Positive self-beliefs → Worry → Prosocial behaviors	0.019	0.022	< 0.001 to < 0.001	0.038
Childhood SES → Social emotion → Worry → Prosocial behaviors	0.162	0.141	< 0.001 to < 0.001	0.019
Childhood SES → Worry → Prosocial behaviors	0.912	0.894	0.089 to < 0.001	0.022

Note. SES, socioeconomic status; *SE*, standard error; BC 95% CI, bias-corrected 95% confidence interval.

**Table 5:** Standardized direct and indirect effects from childhood SES to antisocial behaviors.

Paths	β	SE	BC 95% CI	*p*
Childhood SES → Positive self-beliefs → Antisocial behaviors	-0.942	0.988	-3.392 to 0.842	0.295
Childhood SES → Social emotion → Positive self-beliefs → Antisocial behaviors	-0.118	0.176	-0.989 to 0.063	0.167
Childhood SES → Social emotion →Antisocial behaviors	0.777	0.955	-0.351 to 4.806	0.141
**Childhood SES → Positive self-beliefs → Worry → Antisocial behaviors**	**0.196**	**0.150**	**0.020 to 0.838**	**0.033**
**Childhood SES → Social emotion → Positive self-beliefs → Worry → Antisocial behaviors**	**0.024**	**0.003**	**< 0.001 to < 0.001**	**0.037**
**Childhood SES → Social emotion → Worry → Antisocial behaviors**	**0.206**	**0.169**	**0.040 to < 0.001**	**0.008**
**Childhood SES → Worry → Antisocial behaviors**	**1.164**	**0.863**	**0.165 to 4.618**	**0.016**
Childhood SES → Positive self-beliefs → Antisocial behaviors	0.523	0.628	-0.709 to 1.996	0.452
Childhood SES → Social emotion → Positive self-beliefs → Antisocial behaviors	0.065	0.097	-0.063 to 0.295	0.294
Childhood SES → Social emotion →Antisocial behaviors	0.110	0.486	-0.638 to 1.652	0.649

Note. SES, socioeconomic status; *SE*, standard error; BC 95% CI, bias-corrected 95% confidence interval.

### Pathways from childhood socioeconomic status to prosocial behaviors

Childhood SES exhibited significant effects on prosocial behaviors through worry (*β* = 0.912, *p* = .022), explaining 83.07% of the total effect. Childhood SES exhibited significant effects on prosocial behaviors through social emotions, positive self-beliefs and worry (*β* = 0.019, *p* = .038), explaining 1.73% of the total effect. Childhood SES exhibited significant effects on prosocial behaviors through social emotions and worry (*β* = 0.162, *p* = .019), explaining 14.76% of the total effect. Childhood SES exhibited significant effects on prosocial behaviors through positive self-beliefs and worry (*β* = 0.153, *p* = .041), explaining 14.02% of the total effect.

### Pathways from childhood socioeconomic status to antisocial behaviors

Childhood SES exhibited significant effects on prosocial behaviors through worry (*β* = 1.164, *p* = .016), explaining 81.03% of the total effect. Childhood SES exhibited significant effects on prosocial behaviors through social emotions and worry (*β* = 0.206, *p* = .008), explaining 14.56% of the total effect. Childhood SES exhibited significant effects on prosocial behaviors through positive self-beliefs and worry (*β* = 0.196, *p* = .033), explaining 13.99% of the total effect. Childhood SES exhibited significant effects on prosocial behaviors through social emotions, positive self-beliefs and worry (*β* = 0.024, *p* = .037), explaining 1.72% of the total effect.

## Discussion

To the best of our knowledge, few studies have examined the pathways from childhood SES to prosocial and antisocial rule-breaking behaviors, mediated by positive self-beliefs, social emotions, and worry among Chinese adolescents. Childhood SES significantly influenced prosocial and antisocial behaviors through positive self-beliefs and worry. Childhood SES significantly influenced prosocial and antisocial behaviors through social emotions, positive self-beliefs and worry. Childhood SES significantly influenced prosocial and antisocial behaviors through social emotions and worry. Childhood SES significantly influenced prosocial and antisocial behaviors through worry.

This study showed that childhood SES significantly affected on prosocial and antisocial behaviors through worry. Enhancing children’s prosocial behaviors may also contribute to improvements in adverse well-being outcomes^18^. A study found that stronger prosocial attitudes were correlated with improved well-being across different regions^40^. Among socially anxious undergraduates, engaging in prosocial behaviors was associated with reduced social avoidance^41^. There is growing interest in how worry, as an anxiety-related cognitive process, contributes to the development of paranoid thinking^42^. Individuals with high levels of worry, prosocial behaviors can be increased through interventions such as performing positive behaviors, which may also relate to broader social benefits^43^. Community connection within prosocial interventions influences health outcomes, individual interests, and emotional expression^41^.

This study showed that childhood SES significantly affected on prosocial and antisocial behaviors through positive self-beliefs and worry. Positive self-evaluations of children’s abilities may influence their judgments and responses in prosocial situations^44^. Individual beliefs encompass both positive beliefs on the functionality of worry and negative beliefs of worry itself^45^. Children’s negative self-evaluations are more strongly linked to antisocial behaviors than to prosocial behaviors, reducing the likelihood of positive interactions^46^. Autonomous self-regulation has been associated with greater prosocial behaviors^47^, while self-endorsed functioning is linked to positive psychological adjustment in adolescents^48^. Encouraging children’s self-determination may serve as a crucial additional element in enhancing social-emotional skills and reducing antisocial behaviors^18^. Prosocial interventions increased resilience, reduced social anxiety and negative affect, and improved overall positive emotion^41^. Kindness interventions reduce worry and impact both positive and negative beliefs^43^.

This study showed that childhood SES significantly affected on prosocial and antisocial behaviors through social emotions and worry. Understanding how adolescents develop active prosocial behaviors requires starting with their actions and social interactions^16^. Emotions link actions to the world, drawing adolescents into relationships by fostering interpersonal connections through interest^49^. Children inhabit a world where they engage in mutual responsiveness, constantly addressing and being addressed, within social relationships infused with deep emotions of concern, interest, and enjoyment^50^. The development of social knowledge emerges as social skills, reflecting how children naturally engage in social-emotional relationships with others, where the reality of interpersonal connections is inherent rather than a learned cognitive achievement^16^. A German study indicated that prosocial behaviors negatively predicted emotion dysregulation among adolescents^51^. A Dutch study found that there are significant relationships between adolescents’ social-emotional skills and their prosocial behaviors^52^. Prosocial behaviors may initially be driven not by a concern for others’ welfare, but by social emotional skills that arise from engaging in interactions with others^16^. A Chinese study found that undergraduates’ social-emotional competency requires improvement^53^. Prosocial interventions not only enhanced positive emotions and academic engagement^54^, but also supported that engaging in prosocial behaviors is evolutionary strategy to alleviate distress from emotional rejection and reduce the risk of further social exclusion^55^. Universal, school-based social-emotional skills programs demonstrated positive effects on children’s prosocial behaviors and reduction of antisocial behaviors^56^.

This study showed that childhood SES significantly affected on prosocial and antisocial behaviors through social emotions, positive self-beliefs and worry. Self-beliefs play a pivotal role in determining psychological health, negative self-beliefs, such as, viewing oneself as a failure or unlikeable, are essential in understanding and treating mental health conditions, that are targeted in psychological interventions^13^.Children learn to understand social situations by learning to anticipate both the positive and negative emotional consequences for themselves and others involved^16^. Positive self-evaluations reflect confidence in individual’s abilities and are likely to increase the willingness to engage in prosocial behaviors, negative self-evaluations may contribute to antisocial behaviors in children^57^. Increased acceptance from others boosts self-esteem and fosters positive social emotions and behaviors, while worsening interpersonal problems diminish self-esteem, leading to negative social emotional and behavioral responses^46^. Engaging in prosocial behaviors might offer modest but enduring benefits to social-emotional well-being and mental health, though they showed no significant difference in levels of depression, anxiety, happiness, or the belief^58^. Developing positive self-beliefs in mental health treatment can counterbalance negative self-perceptions, may provide an alternative approach to addressing antisocial behaviors^13^.

### Recommendations

Prosocial interventions are linked to improved health outcomes among vulnerable groups, proving effective in addressing health disparities, with community connectedness playing a key role in facilitating these interventions^41^. Prosocial interventions demonstrate effectiveness in improving mental health^58^, including behaviors of kindness, pay-it-forward gestures, and expressions of kindness^59^. Expressive helping positively impacts distress, partially through increased expression of positive social emotions, aligning with existing theory and research^60^. Children’s progression through various forms of sharing is shaped by their understanding of social-emotional consequences, they are more inclined to share resources when they anticipate the negative emotions of those who are excluded^16^. Interventions targeting social-emotional skills in children, such as those grounded in cognitive-behavioral approaches, foster appropriate prosocial behaviors and prevent the development of antisocial tendencies^61^. Social Emotional Learning (SEL) is widely recognized as a crucial component in educational practices, with social-emotional skills linked to positive developmental trajectories, including enhanced academic performance and protection against negative beliefs^62^. Meta-analysis of SEL interventions have reported small to moderate effect sizes, highlighting: (a) focusing on the enhancement of students’ social-emotional behaviors, (b) promoting a prosocial behaviors enable the application of social-emotional skills in daily interactions, and (c) relying on the prosocial behaviors to prevent disruptive, antisocial, or harmful behaviors^63^. Interventions designed to enhance childhood social-emotional skills overlap with self-determination skills, strengthening social-emotional, and behavioral competencies^18^. School-based programs for children at risk of emotional and behavioral disorders target self-determination related skills, fostering autonomy and intrinsic positive intentions, which in turn improve prosocial behaviors^64^.

### Strength and limitations

Traditional regression models estimate independent direct effects, however, they face methodological challenges in estimating both direct and indirect effects of childhood SES on prosocial and antisocial behaviors, particularly due to potential collinearity with intermediate confounding factors^65^. By incorporating controlled sociodemographic variables as covariates, SEM provides a robust statistical approach for assessing the direct and indirect pathways among multiple variables by integrating confirmatory factor analysis (CFA) with multiple regression analysis^66^. This study used self-report measures; however, previous research indicates that youth self-reports are generally accurate for internalizing experiences^67^. Additionally, when confidentiality is assured, self-reports of externalizing problems in youth have been found to demonstrate good validity. This study was a cross-sectional design, which weakens the robustness of the direct and indirect associations identified. The sample was predominantly female (77%), which may impact the generalizability of the findings. Future studies should aim for a more balanced sample.

## Conclusion

Prosocial and antisocial behaviors that develop during adolescence are likely to persist into adulthood. This study is the first to examine the pathways linking childhood SES to prosocial and antisocial behaviors, with positive self-beliefs, social emotions, and worry acting as mediators among adolescents. This study offers evidence-based strategies for fostering prosocial behaviors by addressing the impact of childhood SES through adjusting positive self-beliefs, social emotions, and worry in upper- and middle-income countries (UMICs).

## Data Availability

The datasets used and/or analysed during the current study available from the corresponding author on reasonable request.

## Abbreviations

CFA: Confirmatory factor analysis
CFI: Comparative Fit Index
CI: Confidence interval
GFI: Goodness of fit index
IFI: Incremental Fit Index
UMIC: Upper- and middle-income country
PARB: Prosocial and antisocial rule-breaking
RMSEA: Root-mean-square error of approximation
SDT: Self-determination theory
SEE: Social–emotional expertise
SEL: Social emotional learning
SEM: The structural equation modeling
SES: Socioeconomic status
RMSEA: Root mean square error of approximation
